# Spinal cord stimulation induces Neurotrophin-3 to improve diabetic foot disease

**DOI:** 10.1007/s00795-024-00410-2

**Published:** 2024-11-17

**Authors:** Yi Liu, XuanPeng Li, HaiWen Xu, Ke Sun, Hui Jun Gong, Cheng Luo

**Affiliations:** 1 Department of Neurosurgery, National Regional Trauma Center, the First Affiliated Hospital of Kunming Medical University, No.295 Xichang Road, Kunming, Yunnan Province China; 2 The Second Department of Neurosurgery, the First Affiliated Hospital of Kunming Medical University, No.295 Xichang Road, Kunming, Yunnan Province China

**Keywords:** Spinal cord stimulation, Neurotrophin, Neurotrophin-3, Diabetic foot disease, Wound healing

## Abstract

Low-extremity ischemic disease is a common complication in diabetic patients, leading to reduced quality of life and potential amputation. This study investigated the therapeutic effect of spinal cord stimulation (SCS) on patients with diabetic foot disease and a rat model of diabetic foot injury. SCS was applied to patients with diabetic foot disease, with clinical assessments performed before and after therapy. Blood levels of NGF, BDNF, and NT-3 were determined by ELISA. A rat model of diabetic foot injury was established to validate NT-3’s role in SCS therapy. SCS therapy improved the condition of patients with diabetic ischemic foot disease and promoted wound healing in the rat model. NT-3 levels significantly increased after SCS therapy in both patients and rats. Recombinant NT-3 administration improved wound healing and re-vascularization in the rat model, while NT-3 neutralization abrogated SCS’s therapeutic effect. SCS improves the condition of patients with diabetic ischemic foot disease by inducing NT-3 production. Both SCS and NT-3 supplementation show therapeutic potential for ameliorating diabetic foot disease.

## Introduction

In recent years, the incidences of lower extremity arterial disease (LEAD) induced by diabetes, arteriosclerosis obliterans of lower limbs, and thromboangiitis obliterans have been increasing year by year [1–3]. Chronic ischemic disease of the lower limbs leads to long-term and persistent limb pain, persistent ulcers, and other complications, which seriously affect the quality of life of patients and may lead to gangrene and amputation [4]. There is an increased risk of chronic limb ischemia in diabetic patients due to peripheral arterial diseases, which could culminate in lower extremity amputation [5]. The chronic pain in the diabetic patients with LEAD also seriously undermine the mobility and self-care ability [6]. Except for pain management, current treatment for diabetes-associated LEAD include open surgical and percutaneous endovascular revascularization therapy [7]. Nevertheless, there is a need for the development of non-invasive treatment approaches for diabetic LEAD.

Neuromodulation involving the application of electricity directly to the neural circuit has emerged as a pain control strategy [8]. Spinal cord stimulation (SCS) has been established as a viable method of neuromodulation in patients suffering from chronic neuropathic pain, which has been practiced for over 50 years with proved effect inf pain relief [9]. SCS relies on the electrodes placed into the epidural space of the spinal canal to stimulating the sensory neurons and the posterior column conduction bundle in the spinal cord through electric current, interrupting the transmission of pain signals [10]. SCS can not only attenuate pain-related signaling pathways and maintain the neurotransmitter balance, but also mitigates inflammation and reduces the production of pain-related neuropeptide to alleviate pain [11]. There is clinicial evidence that electrical SCS could ameliorate painful diabetic peripheral neuropathy [12]. Mounting evidence also suggest that SCS improves the limb salvage and clinical situations in patients with peripheral vascular disease and non-reconstructable critical limb ischemia [13–15]. However, the underlying mechanisms remain elusive.

Neurotrophins are a family of functionally related proteins which regulate neuronal survival, synaptic function and neurotransmitter release in the central and peripheral nervous system of adults [16]. Since the 1950s, these factors have been widely studied in traumatic brain injury and spinal cord injury [17, 18]. For example, cell therapy with sustained delivery of neurotrophic factors has been demonstrated as an effective therapeutic strategy for spinal cord injury [18, 19]. Although there is little evidence that neurotrophic factors are implicated in chronic limb ischemia and LEAD, increasing evidence indicates that neurotrophins, such as nerve growth factor (NGF), play significant roles in wound healing [20]. For instance, NGF enhances fibroblast and keratinocyte proliferation, angiogenesis and myofibroblast differentiation [21–23]. In this study, we evaluated the therapeutic effect of SCS on the patients with diabetic ischemic foot disease and the levels of neurotrophic factors. The rat model of diabetic foot injury was established to validate the role of Neurotrophin-3 (NT-3) as a wound-healing factor for SCS therapy.

## Methods

### Clinical analysis and diagnosis

A total of 10 patients (6 males, 4 females) with diabetic foot disease refractory to conventional treatments were recruited for spinal cord stimulation (SCS) therapy using the Medtronic brand spinal cord stimulator (Rechargeable Implantable Spinal Cord Neurostimulator 97,715). The mean age of the patients was 42.5 ± 7.8 years (range: 32–55 years). All patients had Type 1 diabetes mellitus. The mean duration of diabetes was 18.7 ± 5.6 years (range: 10–28 years). The mean HbA1c level at the time of enrollment was 8.2 ± 1.3% (range: 6.8–10.1%). All patients had at least one foot ulcer (Wagner grade 2–4) that had not responded to standard wound care for a minimum of 8 weeks. The mean duration of the foot ulcer before SCS therapy was 12.8 ± 4.9 months (range: 6–22 months). Comorbidities included hypertension (*n* = 5), dyslipidemia (*n* = 4), and peripheral arterial disease (*n* = 7). All patients had previously undergone conventional treatments including optimal glycemic control, wound care, and revascularization procedures (when applicable) without satisfactory improvement.

The surgical procedure involved a posterior midline incision in the lower thoracic region to implant a stimulation electrode. The electrode was positioned in the epidural space with contacts spanning the T10-T12 vertebral levels. This placement was chosen to target the spinal segments innervating the lower limbs, which is more appropriate for addressing diabetic foot ulcers. Post-operative programming was performed to optimize stimulation parameters, typically using frequencies of 40–100 Hz, pulse widths of 210–480 μs, and currents of 1–6 mA. The Medtronic SCS device allowed for a wider range of parameters, including frequencies up to 1200 Hz, pulse widths up to 1000 μs, and program intensities of 0–100 mA. The treatment protocol consisted of two episodes per day, with each episode lasting 20 min, followed by a 10-min interval. This regimen was designed to provide targeted pain relief while allowing for rest periods between stimulation. Patients or their caregivers were trained in basic programming of the Medtronic device to manage these treatment sessions and adjust settings as needed to enhance treatment efficacy and satisfaction. The peripheral blood samples were collected at different time points after SCS therapy. The use of human sample was approved by the ethics committee of The First Affiliated Hospital of Kunming Medical University University (NO. [2023] L-24). All the patients provided the written informed consent.

To minimize infection risk, we implemented comprehensive preventive measures throughout the treatment process. Patients were carefully screened for active infections, with those having uncontrolled infections excluded. Pre-operative preparation included a thorough cleansing regimen, stabilization of existing ulcers, and optimization of glycemic control. Peri-operatively, prophylactic antibiotics were administered, surgical sites were prepared with chlorhexidine-alcohol solution, and minimally invasive techniques were employed where possible. The stimulation electrode was implanted at the C3 level, away from existing foot ulcers, with watertight closure of incisions. Post-operatively, incision sites were closely monitored, patients were educated on proper wound care and hygiene, and regular follow-ups were scheduled to assess wound healing and detect early signs of infection. These measures aimed to reduce infection risk while providing the potential benefits of SCS therapy to high-risk diabetic patients with foot ulcers.

### ELISA

The relative levels of NGF (Nerve Growth Factor), BDNF (Brain-Derived Neurotrophic Factor), and NT-3 (Neurotrophin-3) in the venous blood samples were determined using corresponding ELISA kits (Elabscience, TX, USA) based on the supplier’s protocol. Briefly, the assay plate was coated with corresponding antibodies at 4 ℃ overnight, and 100 μL of samples or standard was added to each well for 2-h incubation at ambient temperature. After washing, each well was filled with 100 μL of biotin-labeled detection antibody for 1-h incubation, which was followed by the incubation with 50 μl of streptavidin-HRP for another 1 h. After washing, 100 μL of chemiluminescent development reagent was applied in each well for 10 min. The absorbance of each sample was recorded at 450 nm using a microplate reader. The concentration of each molecule was calculated based on the standard curve method. Total protein content in each sample was determined using the Pierce BCA Protein Assay Kit (Thermo Fisher Scientific, Waltham, MA, USA) according to the manufacturer’s instructions. ELISA results were normalized against the protein content in each sample.

### Animal model of diabetic foot injury

Adult female SPF grade SD rats (4–6 weeks, 150–200 g) were purchased from Shanghai Laboratory Animal Center (SLAC) Co., Ltd. (Shanghai, China). The rats were housed in a temperature-controlled environment (22 ± 2 °C), with a 12-h light cycle and free access to food and water. The rats were fasted for 12 h and injected intraperitoneally with a dose of 50 mg/kg Streptozotocin (STZ) prepared in sterile citric acid sodium citrate to induce diabetes. The levels of blood sugar and urine sugar were measured after 2 weeks. The control group was administrated with the same volume of normal saline. When the blood sugar level was above 16.7 mmol/L and the urine-sugar level was above 3 mmol/L, and urine output was doubled, the diabetic model was deemed successfully established [24, 25]. For urine output measurement, rats were individually housed in metabolic cages (Tecniplast, Italy) for 24-h urine collection. Baseline measurements were taken before STZ injection, and follow-up measurements were conducted 14 days post-injection. Urine volume was measured using a graduated cylinder. Urine output was considered doubled when the 24-h volume was at least twice the baseline measurement. Food and water intake were monitored throughout the experiment.

To induce the diabetic foot model, rats were anesthetized with isoflurane (3% for induction, 1.5–2% for maintenance) delivered via a nose cone. Once a surgical plane of anesthesia was confirmed by the absence of a pedal reflex, a round skin wound was created on the back instep of diabetic rats with a 5 mm disposable skin biopsy perforator and Westcott scissors, and the depth of the wound reached the fascia. A similar foot damage was created in the control group without diabetes induction. Throughout the procedure, the rats’ vital signs were monitored, and body temperature was maintained using a heating pad. Post-operative analgesia was provided with subcutaneous buprenorphine (0.05 mg/kg) every 12 h for 48 h. There were 5 animals in each experimental group, including control group, control + foot injury group, STZ induction group, STZ + foot injury group, and other intervention groups (SCS or NT-3 intervention). All the animal protocols were approved by the animal use and care committee of Kunming Medical University hospital (NO. kmmu20231637).

### Blood glucose and urine-glucose testing

The blood glucose level and urine glucose level in each experimental group were detected by the Blood Glucose Content Assay Kit (BC2495, Solarbio, Shanghai, China) and the Rat Urine Sugar (Ug) ELISA Kit (RC-D907037, Ruichuang Biotech, Tianjin, China), respectively.

### Spinal cord stimulation (SCS) therapy and NT-3 intervention

Electrode implantation was conducted 1 week after the foot injury induction. Under under 2% isoflurane anesthetization, the laminectomy was conducted at the vertebral level, with the distal end of the electrode being inserted in the rostral direction. The electrode proximal end was fixed subdermally at the base of the neck, which was connected to an adapter. The adapter was connected to a programmable spinal cord stimulator with a controlling and recording software (Precision Spectra™ Spinal Cord Stimulator System, Boston Scientific, MA, USA). The current amplitude was increased from zero to 4 Hz, 0.25 ms until the muscle contraction was observed in the hind limbs under 2% isoflurane. In treatment group animals, SCS was administrated 1 week after the foot damage in each group for 6-h daily duration, at a frequency of 1,000 Hz, pulse width of 0.1 ms, and using 60% of the motor threshold. The ELISA of different factors was conducted on 1 d、7 d、14 d and 30 d after SCS, and the wound healing and histological analyses were performed on day 30. For NT-3 intervention, the STZ + operation group was administered rat recombinant NTF3 (Qkine, Cambridge, UK) at 1 mg/kg intraperitoneally, twice per week for 4 weeks. To neutralize NT-3, anti-NT-3 antibody (Biosensis, Thebarton, Australia) was injected at the dose of 2 mg/kg intraperitoneally, twice per week for 4 weeks. Control groups received equivalent volumes of saline following the same schedule. All injections were performed under brief isoflurane anesthesia to minimize stress and ensure accurate dosing.

### Immunohistochemical (IHC) staining

Immunohistochemical staining of CD31 was performed on 5-μm sections of tissues at the wound site. After deparaffinization and hydration, the sections underwent heat-induced epitope retrieval in citrate buffer (pH 6.0) for 20 min. Endogenous peroxidase activity was quenched with 3% hydrogen peroxide for 10 min. The sections were then washed three times with PBS and blocked in 5% normal goat serum for 1 hat room temperature. Primary anti-CD31 antibody (Abcam, ab28364, 1:100) was applied and incubated overnight at 4 °C. After washing with PBS, the sections were incubated with biotinylated secondary antibody for 30 min, followed by streptavidin-HRP for 30 min at room temperature. The signal was developed using DAB substrate, and the sections were counterstained with hematoxylin. The slides were dehydrated, cleared, and mounted with permanent mounting medium. IHC images were captured using a Leica DM4000 B LED microscope (Leica, Wetzlar, Germany). CD31-positive vessel density was quantified by counting the number of CD31-positive vessels in five random high-power fields (400 ×) per section using ImageJ software (NIH, Bethesda, MD, USA).

### Hematoxylin and Eosin (H&E) staining

The H&E Stain Kit (ab245880, Abcam, Cambridge, UK) was used for histological analysis. Deparaffinized/hydrated sections were stained with Hematoxylin solution for 5 min. The sections were then rinsed three times with distilled water and further incubated with the Bluing Reagent for 2 min. After washing and dehydration in absolute alcohol, the tissue sections were further stained with Eosin Y Solution for 3 min. The sections were rinsed with absolute ethanol for three times and mounted to a slide, and the stained sections were imaged using a Leica DM2500 upright microscope (Leica Microsystems, Wetzlar, Germany).

### Statistics

All the quantitative data were expressed in mean and standard deviation (SD), and data analysis was performed by GraphPad Prism software (GraphPad Software, NY. USA). Student’s t test was used to compare the difference between two groups, and multiple comparisons were conducted via one-way ANOVA with a defined statistical threshold of P < 0.05.

## Results

### SCS improves the condition of the patients with diabetic ischemic foot disease

**Fig. 1:**
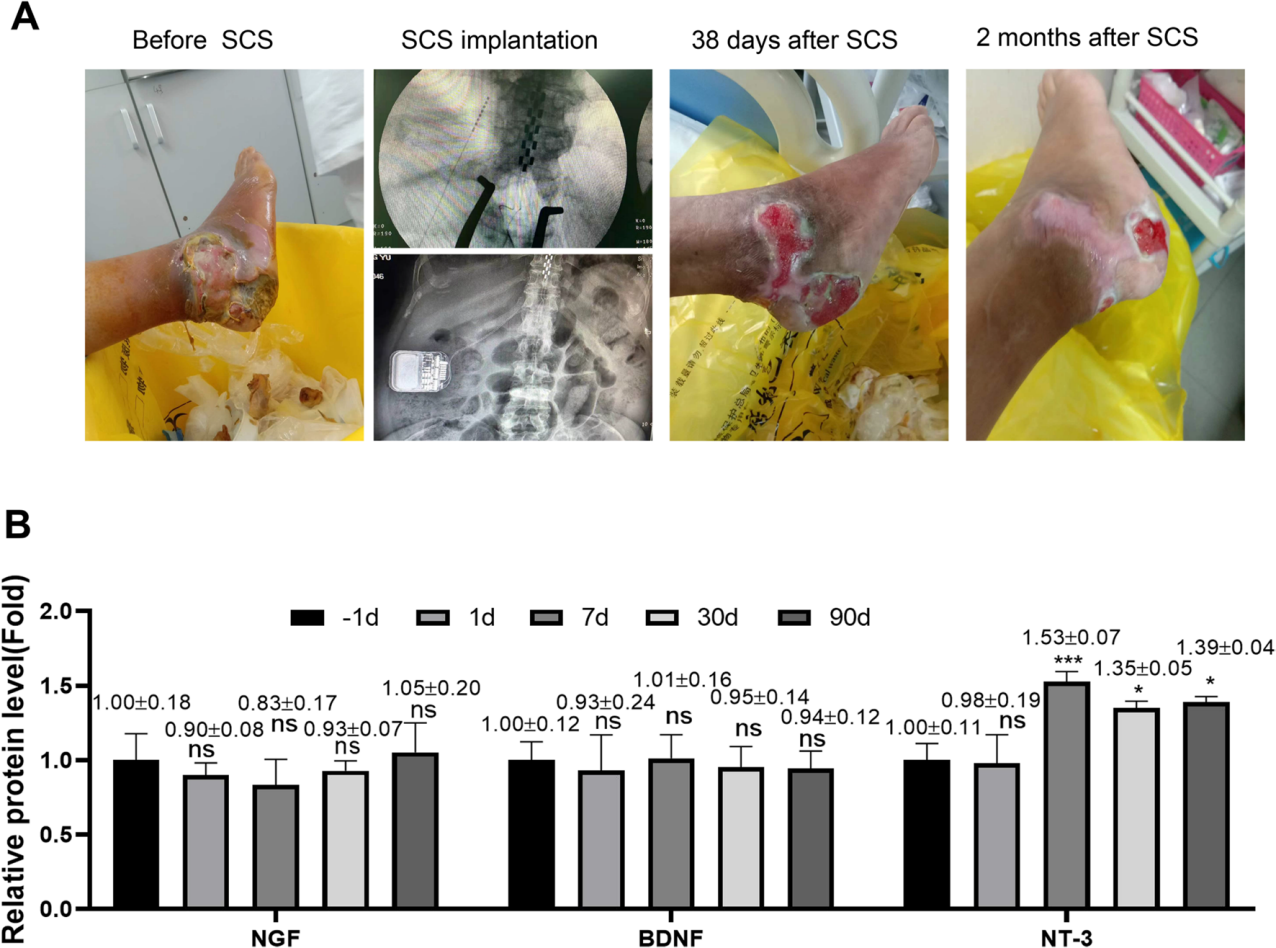
SCS improves the condition of the patients with diabetic ischemic foot disease. **A** Clinical assessment of patients with diabetic ischemic foot disease before and after SCS therapy. X-ray image showing the implanted epidural catheter in the lower thoracic spine (T10-T12 region) for spinal cord stimulation in a patient with diabetic foot ulcers. **B** ELISA analysis of the levels of neurotrophic factors (NGF, BDNF, and NT-3) before and after SCS therapy in the venous blood samples. *P < 0.05, **P < 0.01, ***P < 0.001, ****P < 0.0001

In this study, we recruited 10 patients with diabetic ischemic foot disease, and applied SCS therapy to monitor the beneficial effect. The patients were followed for 2 months and the clinical assessment indicated that SCS therapy gradually improved the conditions of diabetic ischemic foot (Fig. 1A). We also detected the levels of neurotrophic factors (NGF, BDNF, and NT-3) before and after SCS therapy in the venous blood samples by ELISA. BNDF remained unchanged in the course of therapy, and there was a mild increase of NGF after 2 months of SCS therapy. There was a significant elevation of NT-3 after one week therapy, and the NT-3 levels remained at a high level after one and 3 months of therapy (Fig. 1B). These data indicate that increased NT-3 level is associated with the beneficial effect of SCS therapy in the patients with diabetic ischemic foot disease.

### Diabetes induction hinders the foot wound healing and induces the reduction of NT-3 level in the rat model

**Fig. 2:**
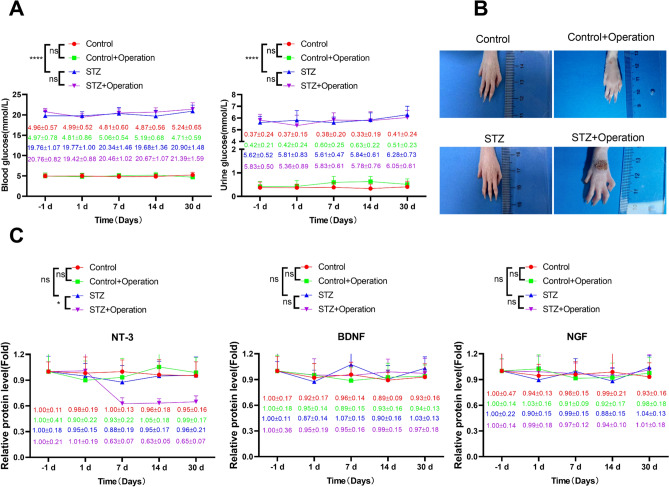
Diabetes induction hinders the foot wound healing and induces the reduction of NT-3 level in the rat model. A rat model of diabetic foot injury was established by streptozotocin (STZ) injection and foot surgical injury operation (STZ + operation). The control group was administrated with normal saline. **A** Detection of blood and urine glucose levels in each experimental group. **B** Representative images of foot wound on day 30 after foot surgical injury operation in each group. **C** The analysis of the neurotrophic factors in the venous blood samples in each group. *N* = 5 in each group. *P < 0.05, **P < 0.01, ***P < 0.001, ****P < 0.0001

To study the potential mechanism of SCS in improving diabetic foot disease, we established a rat model of diabetic foot injury by streptozotocin (STZ) injection and foot surgical injury induction. There was a significant increase of blood and urine glucose levels in the STZ-induced diabetic groups compared to the control group, indicating the successful induction of diabetic model (Fig. 2A). We also followed the wound healing conditions for 30 days in the control and STZ group after foot injury operation. The data showed that in the control group the wound was greatly improved while there was a larger wound remaining in the STZ + operation group (Fig. 2B). The analysis of the neurotrophic factors revealed that NGF and BDNF levels did not show significant changes in all experimental groups. The level of NT-3 also remained stable in the control, control + operation and STZ groups. While in the STZ + operation group, there was significant drop of NT-3 level 7 days after the foot injury induction (Fig. 2C). These data prompted us to further investigate the involvement of NT-3 in diabetic foot injury after SCS therapy.

### SCS improves the wound healing and elevates NT-3 level in the rat model of diabetic foot injury

**Fig. 3:**
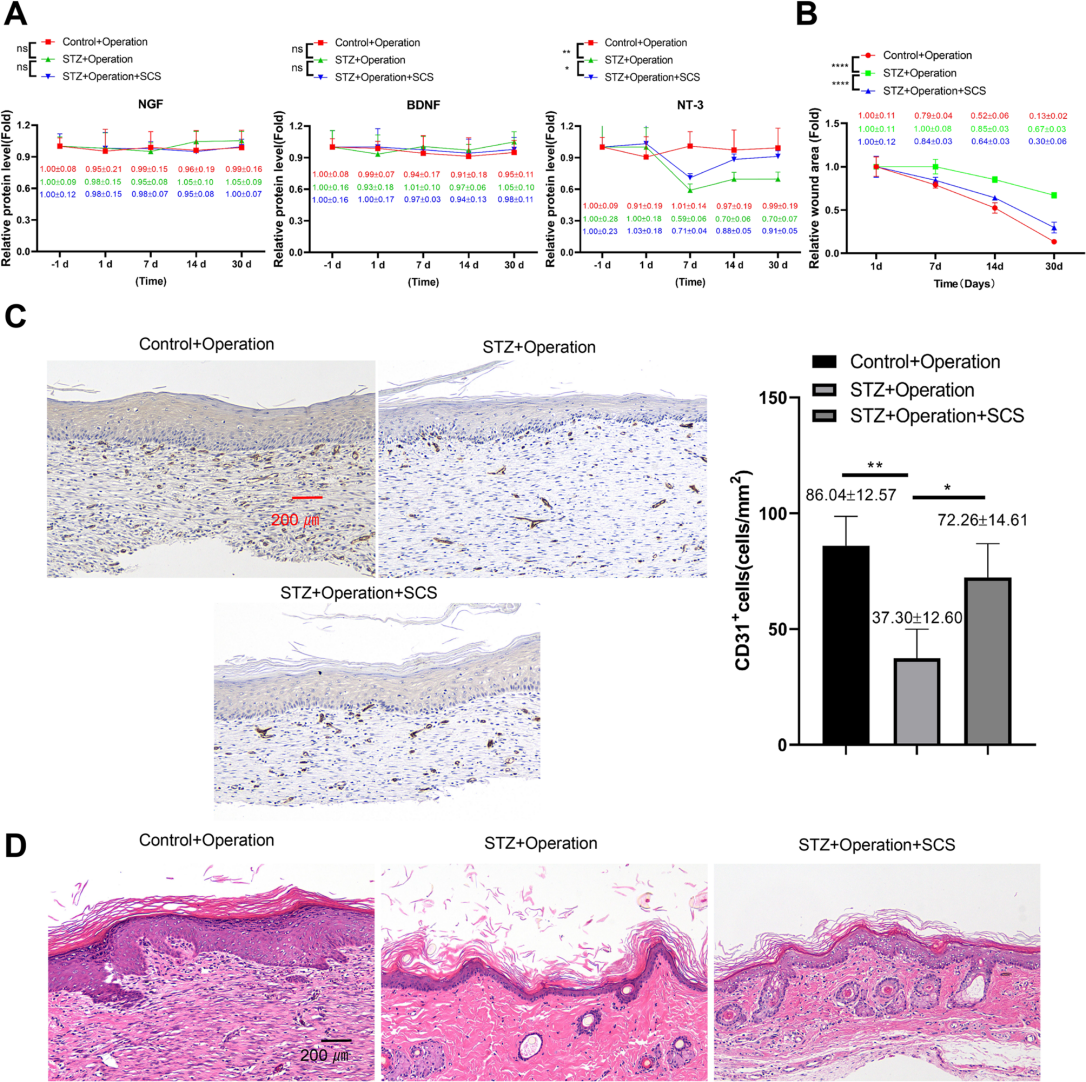
SCS improves the wound healing and elevates NT-3 level in the rat model of diabetic foot injury. The STZ + operation group was provided with SCS therapy for 30 days (STZ + operation + SCS). **A **ELISA analysis of NGF, BDNF and NT-3 levels in the venous blood samples of each experimental group. **B** Representative images of foot wound on day 30 after foot surgical injury operation in each group. **C** IHC staining of the CD31 at the wound site in each experiment group on day 30 after foot surgical injury operation. **D** H&E staining at the wound site in each experiment group on day 30 after foot surgical injury operation. *N* = 5 in each group. *P < 0.05, **P < 0.01, ***P < 0.001, ****P < 0.0001

Next, the STZ + Operation group was provided with SCS therapy for 30 days (STZ + operation + SCS). Compared to the control + operation and STZ + operation groups, there were no significant changes in NGF and BDNF levels during the course of SCS treatment. However, the application of SCS therapy enhanced the level of NT-3 on day 7, and NT-3 reached a comparable level to that before foot injury operation in the STZ + operation + SCS group (Fig. 3A). Besides, the application of SCS therapy after 30 days greatly improved the wound healing in the STZ + operation + SCS group (Fig. 3B). IHC staining of the CD31 at the wound site showed that in the STZ + operation group the vascular density was significantly reduced compared to the control + operation, and the application of of SCS therapy after 30 days increased the vascular density in the STZ + operation + SCS group (Fig. 3C). Further, H&E staining at the wound site also showed an beneficial effect of SCS therapy to reduce the tissue damages in the STZ + operation + SCS group (Fig. 3D). Together, these data demonstrated that SCS therapy could improve the wound healing and increase the level of NT-3 in the rat model of diabetic foot injury.

### NT-3 serves as a wound-healing factor for SCS therapy in the rat model of diabetic foot injury

**Fig. 4:**
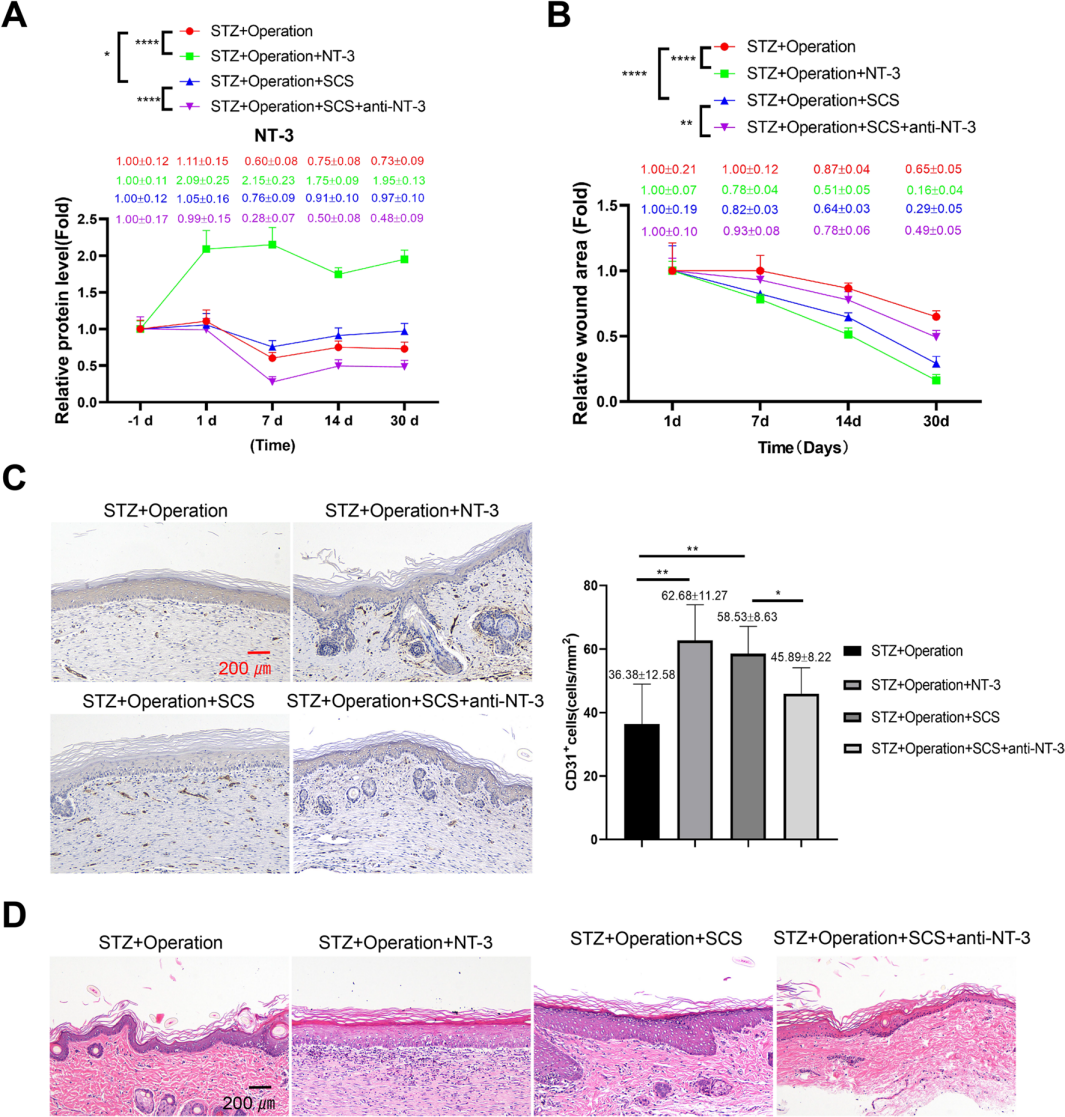
NT-3 serves as a wound-healing factor for SCS therapy in the rat model of diabetic foot injury. To validate the role of NT-3 in the therapeutic effect of SCS treatment, recombinant NT-3 was administrated in the STZ + operation group, and anti-NT3 neutralizing antibody was administrated in the STZ + operation + SCS group. **A** ELISA analysis of NT-3 levels in the venous blood samples of each experimental group. **B** Representative images of foot wound on day 30 after foot surgical injury operation in each group. **C** IHC staining of the CD31 at the wound site in each experiment group on day 30 after foot surgical injury operation. **D** H&E staining at the wound site in each experiment group on day 30 after foot surgical injury operation. *N* = 5 in each group. *P < 0.05, **P < 0.01, ***P < 0.001, ****P < 0.0001

To validate the role of NT-3 in the therapeutic effect of SCS treatment, recombinant NT-3 was administrated in the STZ + operation group, and anti-NT3 neutralizing antibody was administrated in the STZ + operation + SCS group. As expected, the application of recombinant NT-3 significantly increased the level of NT-3 in the venous blood sample when compared to the STZ + operation group, and anti-NT3 neutralizing antibody suppressed the NT-3 levels in the STZ + operation + SCS group (Fig. 4A). The application of recombinant NT-3 greatly enhanced the wound healing in the rat model of diabetic foot injury, and NT-3 neutralization abrogated the therapeutic effect of SCS therapy (Fig. 4B). CD31 staining showed that the application of recombinant NT-3 increased the vascular density at the wound site of the STZ + operation group. In contrast, NT-3 neutralization decreased the vascular density in the STZ + operation + SCS group (Fig. 4C). H&E staining at the wound site further demonstrated the beneficial effect of NT-3 on improving the tissue damages, and the antagonizing effect of NT-3 neutralization on SCS therapy in the STZ + operation + SCS group (Fig. 4D). Therefore, these data suggest that NT-3 serves as a wound-healing factor for SCS therapy in the rat model of diabetic foot injury.

## Discussion

In this study, we reported that SCS improved the condition of the patients with diabetic ischemic foot disease and promoted the wound healing in the rat model of diabetic foot injury. NT-3 levels were significantly elevated after SCS therapy in the diabetic patients and rat model. Besides, the supplementation of recombinant NT-3 improved the wound healing and re-vascularization in the rat model of diabetic foot injury. In contrast, NT-3 neutralization abrogated the therapeutic effect of SCS in the rat model of diabetic foot injury.

Although SCS has shown promising therapeutic effect on traumatic brain and spinal cord injury [26], the underlying mechanisms have not been fully unveiled. A recent study demonstrated that SCS improved ischemia/reperfusion induced sympathoexcitation and ventricular arrhythmias through activating GABA signaling pathways [27]. Extracellular level of GABA in the dorsal horn of neuropathic rats was found to be increased during SCS therapy [28]. These findings suggest the involvement of neurological signaling in SCS therapy. In our study, we showed that SCS therapy could enhance the level of NT-3 in the patients with diabetic ischemic foot disease and in the rat model of diabetic foot injury. Neurotrophins, such as NGF, BDNF and NT-3, play important functions in regulating spinal circuit [29]. The delivery of neurotrophic factors was able to promote axonal growth after spinal cord injury in the adult rats [30]. Emerging evidence indicates that the level of GDNF in cerebrospinal fluid correlates with SCS frequency in patients with neuropathic pain [31]. An early study also revealed that electrical stimulation enhances BDNF expression in spinal cord neurons through ERK-dependent signaling [32]. Our data add novel evidence for the regulation of neurotrophic factor NT-3 by SCS in diabetic ischemic foot disease and injury. We also showed that the delivery of recombinant NT-3 improved the wound injury in the rat model of diabetic foot injury. However, the origin of NT-3 after SCS therapy needs to be clarified, as well as the underlying mechanism by which SCS stimulates NT-3 production.

Neurotrophins have been implicated in re-vascularization in limb ischemic conditions. For instance, NT-3 has been reported as a novel angiogenic factor to induce therapeutic neovascularization in the mouse model of limb ischemia [33]. Gene therapy with NT-3 can promote blood flow recovery to the ischemic foot in the animal model [34]. BDNF overexpression could also increase the capillary density of ischemic muscle and accelerate blood flow recovery in ischemic limb mouse model [35]. Consistent with these findings, we showed that SCS promoted NT-3 level and increased vascular density in the rat model of diabetic foot injury. Therefore, neurotrophins may serve as potential angiogenic factors to improve limb ischemic conditions.

A growing body of evidence pinpoints the wound-healing effect of neurotrophins by promoting angiogenesis, enhancing the function of fibroblasts, inducing keratinocyte proliferation, and triggering myofibroblast differentiation [21–23]. Neuron-derived neurotrophic factor can also modulate endothelial cell function in ischemia-induced revascularization through the activation of Akt/endothelial NOS (eNOS) signaling [36]. NT-3 and its receptor TrkC were found to be expressed in human and mouse skin, with an abundant expression of the receptor in the basal epidermis layer [37, 38]. There is evidence that NT-3 serves as a modulator during morphogenesis and the remodeling of neuroectodermal and mesenchymal interactions in the hair follicles [39]. Furthermore, NT-3 plays a trophic role in sensory neuron generation after skin injury in neonatal rat [40]. Our data also showed that SCS induced the production of NT-3 to enhance wound healing in the rat model of diabetic foot injury. Together, these findings indicate that NT-3 may function as a neurotrophin for epithelial wound healing.

Apart from the effect on NT-3, other mechanisms by which SCS, applied at a distant site, affects ulcer healing in the lower extremities are complex and not fully elucidated. While local effects of SCS on neurotrophic factors are well-documented, the systemic impact is less understood. Several potential mechanisms may explain this distant effect: (1) systemic circulation of neurotrophic factors: SCS-induced increase in neurotrophic factors, particularly NT-3, may enter the systemic circulation, reaching distant sites like foot ulcers [41]. (2) Neuromodulation of autonomic nervous system: SCS could modulate sympathetic outflow, improving microcirculation and tissue oxygenation in the lower extremities [42]. (3) Endocrine effects: SCS might influence endocrine pathways, potentially altering systemic levels of growth factors and inflammatory mediators that affect wound healing [43]. (4) Neuroplasticity: Long-term SCS could induce neuroplastic changes in spinal circuits, indirectly influencing peripheral nerve function and tissue repair processes [44]. (5) Immune modulation: SCS may have systemic effects on immune function, potentially improving the wound healing environment [43]. These hypotheses require further investigation, including detailed analysis of systemic changes in neurotrophic factors, autonomic function assessments, and studies on long-term neuroplastic effects of SCS.

This study has several limitations that should be addressed in future research. First, the sample size of 10 patients in the clinical analysis is relatively small, which may limit the generalizability of the results. A larger cohort study would be beneficial to confirm these findings. Second, while we demonstrated the effects of SCS and NT-3 on wound healing at day 30, we did not perform a time-course analysis of histological changes. Showing the progression of wound healing and vascular changes at multiple time points (e.g., days 7, 14, and 30) could provide valuable information about the dynamics of the healing process and the temporal effects of SCS and NT-3. Furthermore, we did not assess the presence and expression of the corresponding receptor, TrkC, or the receptors for other neurotrophic factors (TrkA for NGF, TrkB for BDNF) in the ulcer tissue. Future study is needed to evaluate the expression of Trk receptors in ulcer tissue, which would provide more detailed mechanistic insights into how neurotrophic factors directly influence the wound healing process.

## Conclusion

In summary, SCS could improve the condition of the patients with diabetic ischemic foot disease and enhance the wound healing in the rat model of diabetic foot injury. SCS induces the production of NT-3, which promotes the re-vascularization and wound healing in the rat model of diabetic foot injury. Our findings suggest that both SCS and NT-3 supplementation may be employed as the therapeutic approach for ameliorating diabetic foot disease.

## Data Availability

The data involved in the study can be found in contact the corresponding author of this article.
